# Putting evolution in elimination: Winning our ongoing battle with evolving malaria mosquitoes and parasites

**DOI:** 10.1111/eva.12530

**Published:** 2017-11-06

**Authors:** Silvie Huijben, Krijn P. Paaijmans

**Affiliations:** ^1^ ISGlobal Barcelona Ctr. Int. Health Res. (CRESIB) Hospital Clínic ‐ Universitat de Barcelona Barcelona Spain; ^2^ Centro de Investigação em Saúde de Manhiça Maputo Mozambique

**Keywords:** drug resistance, evolutionary medicine, insecticide resistance, malaria elimination, resistance management strategies

## Abstract

Since 2000, the world has made significant progress in reducing malaria morbidity and mortality, and several countries in Africa, South America and South‐East Asia are working hard to eliminate the disease. These elimination efforts continue to rely heavily on antimalarial drugs and insecticide‐based interventions, which remain the cornerstones of malaria treatment and prevention. However, resistance has emerged against nearly every antimalarial drug and insecticide that is available. In this review we discuss the evolutionary consequences of the way we currently implement antimalarial interventions, which is leading to resistance and may ultimately lead to control failure, but also how evolutionary principles can be applied to extend the lifespan of current and novel interventions. A greater understanding of the general evolutionary principles that are at the core of emerging resistance is urgently needed if we are to develop improved resistance management strategies with the ultimate goal to achieve a malaria‐free world.

## 1 INTRODUCTION

The Global Technical Strategy for Malaria 2016–2030 envisions a world free of malaria and sets ambitious targets to (i) reduce malaria incidence and mortality rates globally by at least 90% by 2030, (ii) eliminate the disease in at least 35 new countries (as of 2015) and (iii) prevent its re‐establishment in countries that were free of malaria in 2015 (World Health Organization, 2015a). The strategy builds on the tremendous progress in malaria control made since 2000. Aided by the efforts of the Roll Back Malaria initiative and the United Nations Millennium Development Goals (MDGs), infection prevalence in children living in endemic Africa has halved between 2000 and 2015. Of this reduction in malaria burden, 22% and 21% are attributed to artemisinin combination therapies (ACTs), according to Bhatt et al. (2015) and Cibulskis et al. (2016), respectively, and the remainder to vector control interventions (insecticide‐treated nets (or ITNs), later replaced by long‐lasting insecticidal nets (or LLINs), and indoor residual spraying (or IRS) (Bhatt et al., 2015)). Several countries in Africa, South America and South‐East Asia (SEA) are now aiming for malaria elimination. Their elimination efforts continue to rely heavily on antimalarial drugs and insecticides, our front‐line interventions. But will these remain effective as parasites and mosquitoes respond? Recent years have seen malaria detection (Gamboa et al., 2010; Kozycki et al., 2017) and treatment failures (Dondorp et al., 2009; Imwong et al., 2017) due to evolving parasites and alarmingly decreases in insecticide susceptibility due to evolving mosquitoes (Hemingway, Ranson et al., 2016; Ranson & Lissenden, 2016). This so‐called evolutionary arms race (i.e., competition between coevolving species that develop adaptations and counter‐adaptations against each other) between human (host), mosquito (vector) and malaria (parasite) must have occurred since the day they interacted. Evidence for human adaptation to malaria parasites are for instance sickle‐cell and glucose‐6‐phosphate dehydrogenase deficiency (G6PD) genes in certain populations living in highly endemic malaria areas (Luzzatto, Usanga, & Reddy, 1969; Pauling, Itano, Singer, & Wells, 1949).

Only since the discovery about a century ago that the infective agent was a parasite (*Plasmodium*) and that it was being vectored by mosquitoes (*Anopheles*), have we begun to build an artificial arsenal to fight off the parasites and/or their vectors using, for example, drugs, insecticides and larvicides. However, both parasite and vector have evolved a large variety of countermeasures to withstand or endure our interventions. As a consequence, we are now forced to rapidly and continuously design novel drugs and insecticides with different modes of action to replace the failing chemicals. But there are several issues with this so‐called drug/insecticide development treadmill: (i) it is not reactive and by the time resistance is identified, it takes many years before alternatives have successfully replaced a failing chemical, and (ii) it is costly and only a few companies are active in the vector control space, which means only a limited number of new candidates will make it onto the market. We cannot assume that pharmaceutical and chemical industries keep investing in compound development. This may especially be true when we get closer to elimination: when a novel chemistry is needed after significant reductions in disease burden have been achieved, there is almost certainly no financial incentive to investment in product development. And even so, (iii) we cannot assume there will be an infinite amount of novel chemistries to be discovered. Finally, (iv) evolutionary management is predicted to be inherently more effective than speeding up drug discovery (McClure & Day, 2014).

The current situation highlights this problem: at the moment, no new drug is available to replace ACTs when treatment failure becomes a clinical problem. Furthermore, there are no WHO‐approved alternatives to pyrethroid‐based LLINs and the four classes of insecticides for IRS, while resistance to all insecticides is emerging across the globe. This means that we rely heavily—and will continue to do so—on a very limited choice of chemicals, some of which may already be ineffective. The question is as follows: Can we make smarter choices when using our limited set of tools?

In this review, we discuss the evolutionary consequences of the way we currently implement antimalarial interventions and how evolutionary principles can be applied to extend the lifespan of current and novel interventions. The emergence and spread of resistant parasites and mosquitoes is a result of simple Darwinian principles of fitness costs/benefits in the presence/absence of the drug or insecticide. When the failure of malaria interventions is seen as an evolutionary process, that is, the outcome of the competitive interactions between wild‐type (susceptible) and mutant (resistant) organisms, resistance management strategies can be designed to minimize the fitness of mutants, hence slowing down the spread of resistance. Although we focus on current front‐line interventions (insecticides and antimalarial drugs), the concepts apply to all vector and parasite control interventions, which we will discuss at the end of this review.

## 2 ANTIMALARIAL INTERVENTIONS AND THEIR EVOLUTIONARY CONSEQUENCES

**Figure 1:**
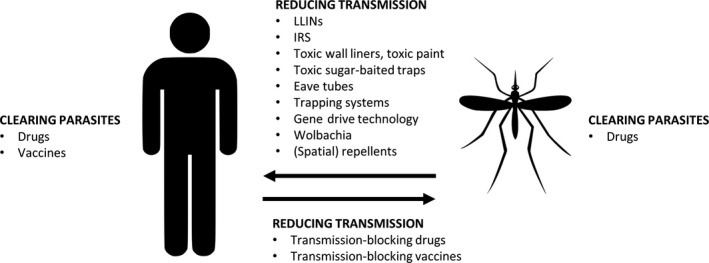
Current and novel interventions aiming to reduce malaria prevalence by either clearing the parasites from the human and/or mosquito or by reducing the transmission probability from mosquito to human or vice versa

Malaria parasites have a variety of different life stages, which can be found in the mosquito vector and in its human host. As such, we target both the mosquito vector (insecticides) and the parasite stages inside the human (drugs), although other alternatives are now being considered and/or in development (Figure 1). Below, we give an overview of the current front‐line intervention strategies for both mosquito and parasite and the current status of the evolutionary adaptations in both organisms.

### 2.1 Targeting the mosquito vector

Reducing the vector population size is an effective method in malaria control. The main front‐line vector control interventions are long‐lasting insecticidal nets (LLINs) and indoor residual spraying (IRS). Since 2000, the distribution of bed nets has been upscaled in the WHO African Region, where in 2015 an estimated 90% of malaria cases and 92% of malaria deaths occurred (World Health Organization, 2016c). Bed nets alone are thought to have averted 450 million clinical cases, which contributes to 68% of overall malaria reduction observed between 2000 and 2015 (Bhatt et al., 2015). To date, pyrethroids are the only insecticide class recommended by the WHO to be used in/on bed nets.

Indoor residual spraying is believed to have prevented roughly 66 million clinical cases, contributing to 13% of the overall reduction in disease prevalence between 2000 and 2015 (Bhatt et al., 2015). Although for IRS three additional classes of insecticides are available (organochlorines, organophosphates and carbamates), the organochlorine DDT was predominantly used during the 50s and 60s as it was cheap and highly effective. More recently, pyrethroids took over the IRS market with approximately 90% of PMI‐funded countries using pyrethroids in their IRS campaigns around 2010 (Oxborough, 2016).

The success of these chemical interventions is related to the quantities being distributed and used: since 2010, close to 1.2 billion pyrethroid‐based bed nets have been distributed, of which 1 billion in sub‐Saharan Africa (Net Mapping Project, 2017). For IRS, the average use between 2000 and 2009, the only period for which we can find data, was 805 metric tons for DDT, 19 for organophosphates, 19 for carbamates and 24 for pyrethroids. An additional 12 tons of pyrethroids was distributed for the (re)treatment of ITNs (van den Berg et al., 2012). Given the switch to pyrethroids in IRS campaigns by the end of this period, combined with increased IRS coverage, it is likely that quantities have risen. Even so, it must be clear that these figures represented a major selection pressure for DDT and, more recently, pyrethroid resistance. Moreover, these numbers exclude insecticide use in the agricultural sector which most likely results in an additional intense selective pressure, particularly in the immature mosquito stages (Birget & Koella, 2015; Chouaïbou et al., 2016).

#### 2.1.1 Detecting insecticide resistance

The WHO has developed bioassays to assess insecticide susceptibility of adult mosquitoes. Young unfed mosquitoes are exposed to a predetermined discriminating dose of an insecticide for an hour, and the percentage mortality in the test population is assessed 24 hr postexposure. However, due to (i) a change in the main resistance mechanism (from the genetic *kdr* mutation to a 1,000‐fold higher pyrethroid resistance due to P450‐mediated metabolic resistance (Toé et al., 2014)) and (ii) the need to better predict the impact of insecticide resistance on malaria control (failure), other methodologies have been introduced to measure the intensity or strength of resistance. The traditional CDC bottle bioassay was updated to a resistance intensity diagnostic test (I‐RDT) by testing susceptibility at four insecticide concentrations (Bagi et al., 2015). The WHO recently also updated its guidelines for insecticide susceptibility testing to include different exposure doses (World Health Organization, 2016b).

Biochemical and/or molecular methods to detect resistance at a mechanistic level can also be powerful tools for screening vector populations. But while the genetic changes of resistant vectors are increasingly being mapped, the correlation between molecular markers and phenotypic assays is not clear, and therefore, there is no consensus whether molecular markers can replace insecticide susceptibility bioassays. In addition, molecular markers continue to evolve; hence, for now, bioassays are gold standard for insecticide resistance surveillance (Weetman & Donnelly, 2015; See also Sternberg and Thomas, 2017).

#### 2.1.2 The emergence and spread of insecticide resistance

The dramatic increase in the prevalence and strength of insecticide resistance that is observed across Africa (Hemingway, Ranson et al., 2016; Ranson & Lissenden, 2016) is likely due to applying single‐class insecticides in public health. The use of a single insecticide in IRS campaigns (DDT in the 50s and 60s; pyrethroids up to very recent) or a single chemical class (only pyrethroids in/on ITNs/LLINs) resulted in selective pressure in the vector population. But selective pressure is also coming from the agricultural sector, given that significantly larger amounts of insecticides are used in food production (A. S. Hien et al., 2017; Reid & McKenzie, 2016) and may act as larvicide which likely leads to higher selective pressure (Birget & Koella, 2015) (Sternberg and Thomas, 2017).

**Figure 2:**
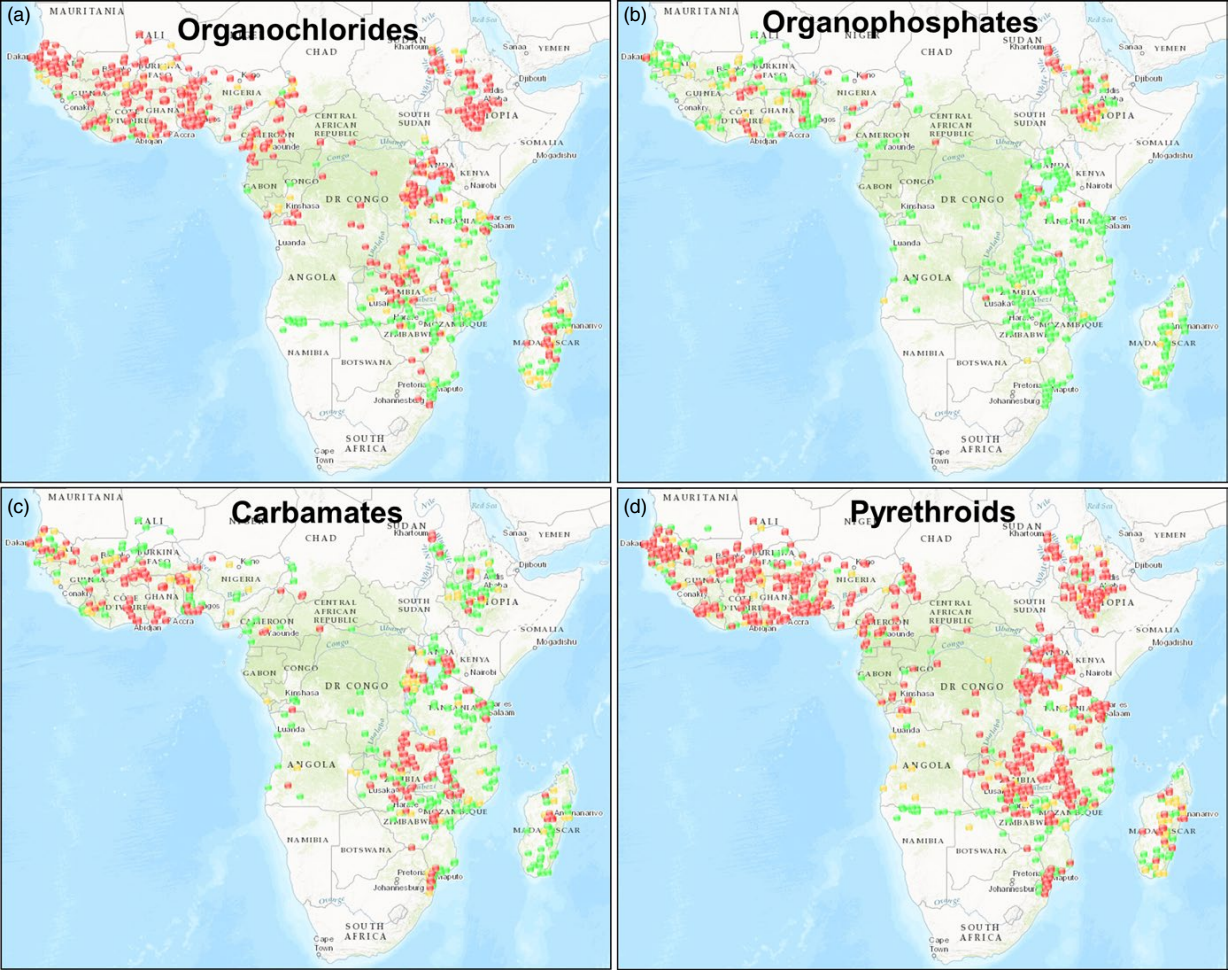
Resistance to the insecticide classes organochlorides (a), organophosphates (b), carbamates (c) and pyrethroids (d) in *Anopheles* species, reported between 2000 and present day (IRmapper.com, assessed 14 June 2017). Reported cases (based on WHO susceptibility tests and CDC bottle assays). Red dots: confirmed resistance (<90% mortality), yellow dots: possible resistance (90%–97% mortality), green dots: susceptibility (98%–100% mortality)

When and where resistance exactly emerged is mostly unknown, as in the past susceptibility was not monitored at the required spatial and temporal scales due to resource and human capacity limitations (Coleman et al., 2017). The initial wave of pyrethroid resistance is likely a reselection of an old DDT resistance mechanism in *An. gambiae* (Hemingway, 2014), as both insecticides have a similar mode of action. As the mutations involved have arisen multiple times and have spread from different locations, we refer to West or East African forms of *kdr*, indicating where they were first detected (Pinto et al., 2007). The cytochrome P450‐based mechanism in *An. funestus* that led to a > 1,000‐fold increase in resistance to pyrethroids was first reported in the Kwazulu‐Natal Province of South Africa (Hargreaves et al., 2000; Wondji et al., 2009). It was probably observed early in this area due to the fact that South Africa already shifted to pyrethroids for indoor spraying in 1996 (Corbel & N′Guessan, 2013), but can be found in multiple sites across Africa nowadays (Barnes et al., 2017; Nwane et al., 2013; Sangba et al., 2016). Alarmingly, there are now countries reporting resistance to three (Djouaka, Atoyebi, et al., 2016; Djouaka, Riveron, et al., 2016; Menze et al., 2016; Olé Sangba et al., 2017; Riveron et al., 2015, 2016) or all four classes of insecticides that we currently have at our disposal (Cisse et al., 2015; Edi, Koudou, Jones, Weetman, & Ranson, 2012) (Figure 2).

#### 2.1.3 The impact of insecticide resistance on malaria transmission

The extent to which the observed widespread and high levels of insecticide resistance threaten malaria control and elimination efforts remains unclear (Rivero, Vézilier, Weill, Read, & Gandon, 2010; Strode, Donegan, Garner, Enayati, & Hemingway, 2014; Thomas & Read, 2016). A recently concluded multicountry assessment to determine the impact of insecticide resistance on the protective effectiveness of LLINs, and thus on malaria transmission, showed there was no evidence of an association between malaria disease burden and pyrethroid resistance across locations (World Health Organization, 2016a). There are, however, several reasons why it may be difficult to observe an impact of resistance:

1. Susceptibility tests: Insecticide susceptibility as determined with standard bioassays may not reflect susceptibility under actual field conditions as such tests are performed with young (2‐ to 5‐day‐old) female mosquitoes following single, limited‐time exposure to an insecticide under constant insectary conditions. As a result, the effect of natural mosquito traits such as sex, age, blood‐feeding status and circadian rhythm (Kulma, Saddler, & Koella, 2013; Oliver & Brooke, 2014) but also climatic variables (Glunt, Blanford, & Paaijmans, 2013) on the toxicity of insecticides is not captured, neither are sublethal effects on blood‐feeding and host‐seeking factors (Glunt et al., unpublished), infection with entomopathogens such as Plasmodium (Alout et al., 2016), or delayed mortality (Viana, Hughes, Matthiopoulos, Ranson, & Ferguson, 2016).
2. Vector species: Resistance is typically characterized for a few major malaria vectors in a given area, but there may be several other malaria vectors present. Although we have always assumed that there are roughly 30‐40 malaria vectors worldwide, recently molecular tools show us we may be dealing with a larger diversity of vector species as well as population diversity within one species (Lobo et al., 2015).
3. Behavioural changes: Apart from the conventional resistance mechanism (target site, metabolic or cuticular resistance), vectors that can avoid contact with insecticides have a clear selective advantage. Several vector species have rapidly shifted their peak biting times to the early evening or late morning, when humans are active but not yet protected by LLINs (Bayoh et al., 2014; Moiroux et al., 2012; Reddy et al., 2011; Russell et al., 2011; Sougoufara et al., 2014; Taylor, 1975) or changed their host preference (Lefèvre et al., 2009).
4. Parasite–mosquito interactions: There may be several ways in which insecticide resistance impacts parasite survival inside the mosquito hosts. Pyrethroid resistance has been demonstrated to reduce mosquito survival following a Plasmodium infection, possibly because the insecticide‐resistant alleles, or closely linked alleles, interfere with the immune system (Alout et al., 2016). In addition, field‐collected *kdr* homozygous mosquitoes that were experimentally infected with malaria and subsequently exposed to deltamethrin impregnated bed nets showed a lower prevalence and lower intensity of infection compared to infected mosquitoes exposed to untreated bed nets. The deployment of resistance mechanisms by the mosquito could potentially be toxic to the parasite (Kristan et al., 2016). On the other hand, *kdr* mutations have been correlated with increased sporozoite prevalence, both in laboratory setting (Alout et al., 2013, 2014) and in the field (Kabula et al., 2016; Ndiath et al., 2014). As such, the overall effect of insecticide resistance on parasite transmission is still unclear, yet these studies highlight that vector control and parasite epidemiology cannot be seen independently.

### 2.2 Targeting the malaria parasite

Malaria parasites likely have a higher potential of adaptation than mosquitoes due to their larger effective population size and shorter generation time. In addition, they are haploid for the majority of their lifecycle, and as a result, beneficial mutations are expressed and can instantly be selected upon (Gerstein, Cleathero, Mandegar, & Otto, 2011). Moreover, the *P. falciparum* genome has, compared to other eukaryotes, an unusual level of genomic plasticity due to systematic mutational biases. This leads to high rates of insertions and deletions which give falciparum parasites an incredible ability to adapt to novel (drug) environments (Hamilton et al., 2017; Miles et al., 2016). Thus, while resistance evolution is always a concern in treatable infectious diseases, this may even be more so in the case of falciparum malaria.

In the absence of an effective malaria vaccine, drugs are used to clear the parasites from the human host (clinical cure, which includes prophylaxis) or to block transmission (from human to vector by targeting, e.g., only the sexual stages that are transmitted to mosquitoes, such as the gametocytocidal drug primaquine), see Figure 1. Several antimalarial drugs have been rolled out over the past 6 decades, from chloroquine shortly after the World War II to artemisinin combination therapies (ACTs) in the 2000s, which is an artemisinin‐derivative drug combined with another antimalarial (either SP, mefloquine, piperaquine, lumefantrine or amodiaquine) (World Health Organization, 2015b). In 2010, the number of ACT courses that were rolled out was 187 million. This number peaked to nearly 400 million courses in 2013 but has since decreased a little due to decreasing global malaria rates (World Health Organization, 2016c).

Another issue to keep in mind is that additional drug strategies have been developed with the aim to accelerate parasite clearance in our effort to eliminate malaria. Several strategies aim to roll out drugs to larger groups of people: mass drug administrations (MDA) or mass screening and treating strategies (MSAT) target all individuals with parasites whether they have symptoms or not (note that MDA also targets the uninfected population, as there is no screening for parasites). In addition, increased efforts to improve malaria surveillance systems (World Health Organization, 2017) will result in more malaria cases being detected. These approaches will lead to increased parasite–drug interactions and thus increased selective pressure on the parasites.

#### 2.2.1 Detecting drug resistance

The time between the introduction of a new drug and clinical resistance to it, which is defined as >10% treatment failure, is the effective lifespan of the drug. Key for resistance management is early resistance detection. The foundation for this is laid by the collaborative platform Worldwide Antimalarial Resistance Network (WWARN) which collects data on antimalarial efficacy (Karunajeewa, 2015). The gold standard for detecting treatment failure is the in vivo assay of parasite–drug response in malaria patients, with a follow‐up time of at least 28 days (World Health Organization, 2015b). The main problem with this approach is that “failure” could be caused by malaria recrudescence (or relapse) or reinfection, which is likely to occur in endemic areas. The molecular methods that are used to distinguish between these categories are not sensitive enough (Juliano, Gadalla, Sutherland, & Meshnick, 2010). Novel and more sensitive methods, such as next‐generation sequencing to determine the genotype composition in the pretreatment sample and the recurring sample, may resolve part of the issue, but this method is expensive and requires expertise. An alternative to in vivo assays are ex vivo assays to test drug sensitivity from patient isolates in laboratory bioassays. However, these assays also require expertise and proximity to good laboratory facilities. As a result, a method that is frequently used is the molecular detection of known resistance markers, often by simple PCR‐RFLP. The advantage of this method is that blood samples can be taken from any site and stored for later analysis in any facility. However, this technique (i) relies on known resistance polymorphisms and will not identify novel unidentified mutations, and (ii) like with insecticide resistance, the link between a resistance marker and drug efficacy is not always very clear (Picot et al., 2009; Volkman, Herman, Lukens, & Hartl, 2017).

#### 2.2.2 The emergence and spread of drug resistance

Resistance of *P. falciparum* parasites to chloroquine, the first widely available antimalarial, started to spread across the majority of malaria‐endemic areas in the 60s and 70s. The drug was replaced with sulphadoxine–pyrimethamine (SP) as the first‐line treatment in several countries in the 90s. SP was used as clinical treatment, but also as intermittent preventive treatment during pregnancy (IPTp) and infancy (IPTi). After resistance to SP appeared in East Africa, it spread very rapidly across Africa in the 90s and 2000s (Naidoo & Roper, 2010; Nair et al., 2003). IPTi‐SP is abandoned in all areas with high levels of resistance (defined as a prevalence of the *Pfdhps* 540 mutation of >50%), but IPTp with SP is still being recommended (World Health Organization, 2015b) and remains successful to date despite the high levels of resistance to SP in some areas (Desai et al., 2016; Walker, Floyd, Ter Kuile, & Cairns, 2017). Soon after the millennium, both chloroquine and SP were replaced with ACTs (World Health Organization, 2015b). In 2009, slower parasite clearing rates by ACT treatment were reported in South‐East Asia (Dondorp et al., 2009), and a few years later, the ACT treatment dihydroartemisinin–piperaquine (DHA–PPQ) was failing in the Greater Mekong Subregion (GMS) (Leang et al., 2013; Saunders, Vanachayangkul, & Lon, 2014). The distribution and spread of the different emerging mutants responsible for artemisinin resistance were almost being tracked in real time. Initially, multiple independent appearances of mutant *PfKelch13* alleles were observed. These now appear to be outcompeted by the—presumably—fitter *PfKelch13* C580Y parasite lineage (Imwong et al., 2017). One case of reduced parasite clearance following ACT treatment with a novel mutation in the *kelch13* gene has also been reported in Africa (Lu et al., 2017). The potential emergence or spread of artemisinin resistance to this part of the world is very worrying.

**Figure 3:**
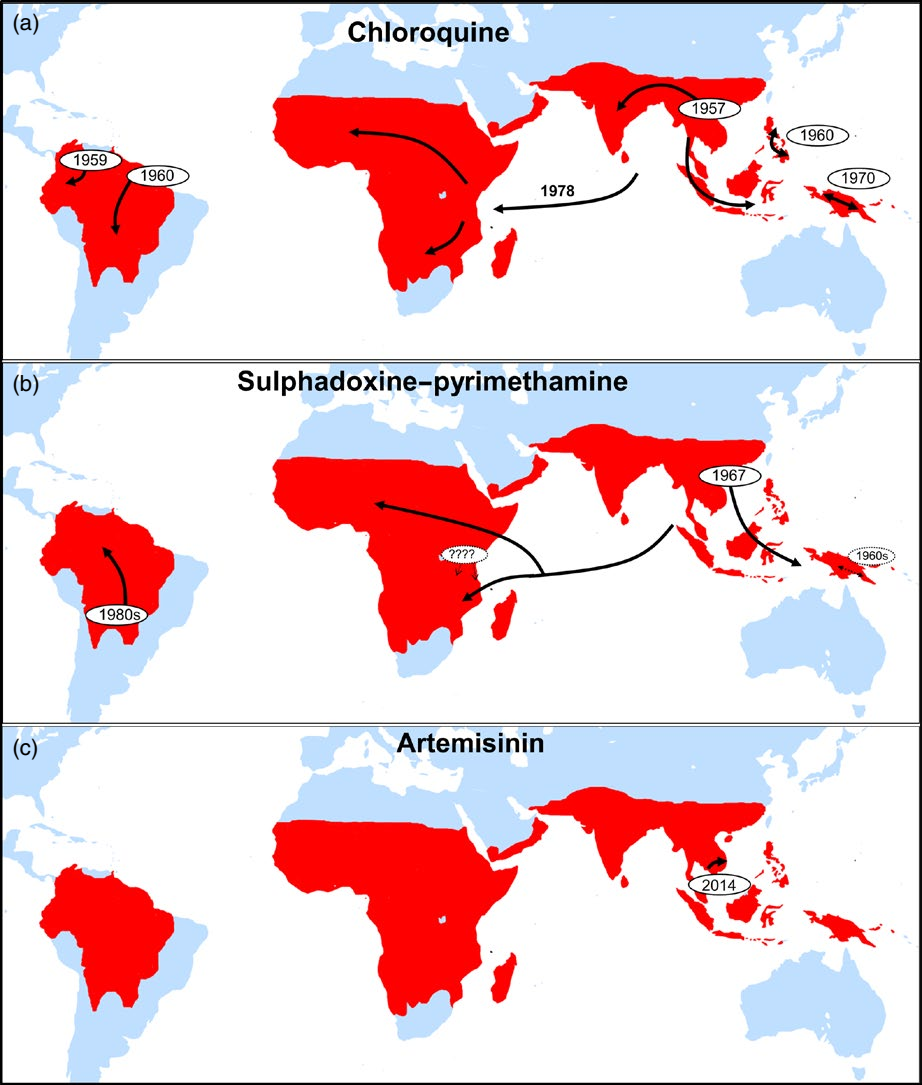
Selective sweeps of chloroquine‐ (a), pyrimethamine–sulphadoxine‐ (b) and artemisinin (*pfKelch13* C580Y lineage)‐ (c) resistant mutants inferred from molecular evolution studies. SP resistance may have several local origins in Kenya (denoted by “????”), but the majority of *dhfr* SP‐resistant infections are a consequence of a selective sweep from a single origin in South‐East Asia. Figure 2a,b is redrawn from Read & Huijben, 2009; and Figure 2c is redrawn from Imwong et al., 2017

However, although falciparum parasites have developed resistance to nearly every available drug, ACTs still remain effective in most parts of the endemic world and have proven to be rather resilient against resistance evolution by not losing their efficacy as rapidly as chloroquine and SP did in the past (Figure 3). This success may be attributed to drug properties, as artemisinins (i) act more rapidly and have a shorter half‐life than all other antimalarials, resulting in a shorter window of selection (Corey et al., 2016), and/or (ii) are protected by a partner drug with a longer half‐life, so selective pressure is always for two drugs of different mode of action at the same time. However, despite this theory, resistance to artemisinin arose before resistance to the partner drug in ACTs (Imwong et al., 2017), possibly due to the history of monotherapy in the area.

A highly curious observation in the evolution of antimalarial resistance is that the current circulating mutants, which have led independently to resistance to a variety of drugs, seem to have gone through the exact same process at the exact same location: the mutant parasites that are found in Africa and that are resistant against chloroquine or SP originate from the GMS and spread in a “hard” selective sweep across the African continent (Nair et al., 2003; Wootton et al., 2002). The fundamental question is as follows: What makes the GMS a breeding ground for resistance evolution? Are the factors related to the host, the parasite, drug practices or are other epidemiological factors such as transmission intensity responsible? A better understanding of this resistance breeding ground is critical as it (i) may provide tools to prevent or delay the onset of resistance for future novel compounds introduced in the GMS, as well as (ii) prevent other areas from becoming resistance hot spots, particularly if the origin lies in epidemiological factors such as low transmission settings (relevant in the context of malaria elimination).

#### 2.2.3 The direct impact of drug resistance on parasite fitness

The rapid pace with which chloroquine‐ and SP‐resistant parasites have spread across the world shows the selective advantage that resistant mutants have in malaria‐endemic areas under enormous selective pressures (exposure to large quantities of drugs). However, a cost of resistance may become apparent in the absence of drug exposure. This has been shown in several—unintended—natural experiments that monitored drug resistance in the years following changes in first‐line treatment. Several years after chloroquine use was abandoned, resistant mutants were only observed at a low frequency or even completely absent (e.g., Laufer et al., 2010; Mekonnen et al., 2014; Mwanza et al., 2016). A recent clinical trial with chloroquine in asymptomatic adults in Mozambique demonstrated that chloroquine may actually be effective again in curing infected patients (Galatas et al., 2017). However, similar patterns have not been observed for SP resistance despite a reduction in drug use (Artimovich et al., 2015; Kateera et al., 2016). The lack of a reduction in frequency of folate resistance markers under the reduced selection pressure is striking, although SP is still used as preventive treatment in pregnant women (IPTp). Nevertheless, Artimovich et al. (2015) even observed an increase in the frequency of triple *pfdhps* mutants during a period of reduced SP pressure. One may conclude that chloroquine‐resistant parasites perhaps carry a higher fitness cost that their SP‐resistant siblings, although direct evidence is lacking.

## 3 APPLICATION OF EVOLUTIONARY PRINCIPLES TO MALARIA CONTROL AND ELIMINATION

The enormous adaptive ability of both *P. falciparum* parasites and their *Anopheles* vector, combined with our dependence on a limited variety of insecticides and drugs, means we can only successfully eliminate malaria if we are able to mitigate resistance evolution. History shows that resistance evolution is nearly always a question of “when,” not “if.” As (i) drugs and insecticides continue to be a cornerstone of malaria elimination strategies, (ii) we only have a few options (ACT with six partner drugs and four insecticide classes with only two different modes of action), which (iii) start to fail, or have already lost efficacy, and (iv) it will take several more years for novel drugs and insecticides to become available (Hemingway, Shretta et al., 2016), the evolutionary management of resistance will be a critical component (McClure & Day, 2014). The main question is whether resistance evolution can be prevented or at the least be significantly retarded with novel approaches. For that, we first need to improve our understanding of the evolutionary dynamics of emergence and spread of resistance and ultimately integrate evolutionary biology and ecology in order to develop appropriate resistance management strategies. A fundamental advantage of the human species is the ability to anticipate and innovate. Evolving populations of mosquitoes and parasites adapt to the current environment, but cannot predict and anticipate future conditions. Human beings, on the other hand, can anticipate evolution and apply knowledge of Darwinian dynamics to create an environment to which mosquitoes and parasites have more difficulty to adapt. But is it possible to cheat evolution? And how?

Understanding fitness costs arising from expressing, utilizing and maintaining molecular or metabolic pathways of resistance will be essential. While these costs fall short by the survival advantage in the presence of the toxic compound (insecticide or antimalarial drug), in the absence of treatment this cost is likely unaffordable and may result in mutant parasites/mosquitoes being outcompeted by fitter ones (Huijben et al., 2010, 2013) (but see *kdr* resistance allele conferring a fitness advantage even in the absence of insecticide exposure (Haoués Alout et al., 2016) and the increase in the mutant frequency under reduced SP pressure (Artimovich et al., 2015)). In other words, mutant parasites and mosquitoes only spread when there is sufficient chemical pressure and will reduce in frequency in the absence of this pressure if the associated fitness cost is sufficiently high. As this cost of resistance is the Achilles heel of resistant parasite and mosquito populations, for effective resistance management strategies, we need to create an environmental context where the benefit of resistance is low and the fitness cost high.

### 3.1 Insecticide resistance management strategies

The WHO set out clear guidelines to manage insecticide resistance in their Global Plan for Insecticide Resistance Management (World Health Organization, 2012a). They urged to implement strategies such as mosaic, rotational or mixture spraying regimes (IRS), which all avoid using the same class of chemical for longer periods of time, hence reducing the selective pressure of resistance against one particular chemical. At least two insecticides with different modes of action are used in different but neighbouring areas (mosaic), rotated from one year to the next (rotation) or are mixed into a single product or formulation and applied (mixture). This approach assumes that if resistance to one insecticide is rare, then the emergence of resistance to multiple insecticides will be extremely rare. A fourth option is to combine two (or more) insecticide‐based interventions with different modes of action, such as IRS in combination with LLINs (Kleinschmidt et al., 2009; World Health Organization, 2014), adulticiding with larviciding (Koella, Lynch, Thomas, & Read, 2009), LLINs with attractive toxic sugar baits (Stone, Chitnis, & Gross, 2016) or LLINs with mosquitocidal ivermectin in humans or domesticated animals (Pooda et al., 2015; Richards, 2017). Such an approach would be particularly effective if one such insecticide would force evolution in a direction that would make mosquitoes more susceptible to the second insecticide (Koella et al., 2009; Volkman et al., 2017). The disadvantage of the approaches described above is that different interventions in the same package target different mosquito behaviours (e.g., biting, resting and breeding behaviours) and may also target different vector species. As such, vectors may only get exposure to one intervention. A fifth approach is the incorporation of refugia, a practice very common in agriculture (Vacher, Bourguet, Rousset, Chevillon, & Hochberg, 2003), which are untreated “pockets” of organisms that do not experience selection for resistance, and serve as a reservoir for insecticide‐susceptible wild‐type organisms (Lenormand, Guillemaud, Bourguet, & Raymond, 1998; Lenormand, Bourguet, Guillemaud, & Raymond, 1999; Park, Haven, Kaplan, & Gandon, 2015). The approaches above (different resistance management strategies or the combination of different insecticide‐based interventions across space and/or time) are not novel as they have for long been used in agriculture to keep herbicide (weeds) and insecticide (pests) resistance under control (Thomas et al., 2012; Sternberg and Thomas, 2017).

Although we will discuss drug‐based interventions below, we like to highlight one pressing issue when it comes to insecticide applications: in the field of medicine, mixing of chemicals is common practice to deter resistance evolution, with, for example, combination therapy being the norm for malaria, HIV and tuberculosis treatment. Monotherapy is nowadays considered wrong, at least at the policy level, but this thinking has surprisingly not yet made its way into the vector control community. Although the pipeline of the Innovative Vector Control Consortium (the IVCC, a product‐development partnership) now includes LLINs treated with two classes of insecticides, one of the insecticides is still a pyrethroid, to which resistance is widespread. In addition, there are no such combination products being developed for IRS.

While it is obvious that mono‐application of insecticides will pave the path for rapid evolution of resistance, it is not at all clear which of the above resistance management strategies should be deployed to maximally deter resistance evolution. What is even less clear is how to determine the optimal deployment conditions (REX Consortium, 2013): How frequently should insecticides be rotated? What spatial scale is needed for mosaic application? The answer to these questions lies in understanding the ecology and evolution of resistant mosquitoes: What are the fitness costs/benefits in the presence/absence of insecticide exposure and what are the patterns of mosquito gene flow? These parameters are likely to differ between (classes of) insecticides, different environments and different vector species, meaning that one size does not fit all when it comes to resistance management. But the key requirement for any strategy to work is to select costly resistance mutations that have a selective disadvantage in the absence the insecticide.

Mathematical models will be of great value to predict the efficacy of the various resistance management strategies, especially as large‐scale field trials whereby different strategies are evaluated will be expensive and require years of testing, a luxury we do not have. A recent model predicts that insecticide mixtures are a favoured resistance management strategy when insecticide effectiveness is high and insecticide exposure low. If the insecticides do not reliably kill sensitive vectors, sequential deployment appears to be a more robust strategy (Levick, South, & Hastings, 2017). In medicine (see below), modelling suggests that higher levels of heterogeneity of selection are associated with longer‐term sustainability of pathogen control (Débarre, Lenormand, & Gandon, 2009), although many of the relevant specific biological parameters are unknown.

Some field data are available on the efficacy of such resistance management. One field trial in Mexico showed that larger‐scale mosaics were effective for the management of pyrethroid resistance in *An. albimanus* (Hemingway et al., 1997; Penilla et al., 1998). Other correlational evidence comes from a multicountry evaluation on the impact of resistance on malaria vector control, in which pyrethroid resistance evolved at a slower rate in locations with pyrethroid LLINs plus non‐pyrethroid IRS than in areas with pyrethroid LLINs alone (World Health Organization, 2016a). However, the selection of an optimal treatment regime will not solely depend on best resistance management practices, but needs to consider resource availability, practicality and a cost‐benefit analysis (Hemingway et al., 2013; World Health Organization, 2010, 2012a).

Over the past few years, evolutionary biologists have started to embrace the question whether “evolution proofing” of chemical compounds is possible, rather than simply combining existing insecticides in different ways. Several novel approaches include new designs or the alternative usage of insecticides, all with the aim to extend the useful lifespan of insecticides.

Read, Lynch, and Thomas (2009) proposed to exploit the biology of the malaria parasites within the mosquito vector. As parasite development time, from mosquito infection to a mosquito becoming infectious, takes roughly 10–14 days, only older mosquitoes need to be targeted as they are responsible for malaria transmission (Koella et al., 2009; Read et al., 2009). Since by that time the mosquito will have completed most of its reproductive lifespan, the selective pressure will be greatly reduced. One way to create a so‐called late‐life‐acting intervention is to use lower concentrations of existing insecticides to only kill the older and weaker mosquitoes (Glunt, Thomas, & Read, 2011). This idea was derived from previous work on delayed‐acting fungal biopesticides (Lynch, Grimm, Thomas, & Read, 2012) and is also the theory behind late‐life‐acting microsporidia to kill vector species (Lorenz & Koella, 2011; see also Sternberg and Thomas, 2017). In contrast, instead of avoiding evolution, one could enforce evolution, but to our advantage. As only few mosquitoes survive to an old enough age for malaria transmission, only a small reduction in survival will remove a significant amount of infectious mosquitoes. This could be achieved if selective pressure is acting on mosquitoes to invest in short‐term reproduction instead of long‐term survival. Such life history shift could greatly reduce malaria transmission (Ferguson et al., 2012), though of course, an evolutionary response in the parasite population, for instance an increased developmental rate with any possible consequences for parasite virulence or transmission, should be anticipated.

Transmission blocking approaches within the mosquito vector avoid the evolution of insecticide resistance using compounds that only target the development of the parasite within the mosquito, hence not putting pressure on the mosquito itself. Childs and colleagues identified a compound that manipulates the steroid hormone 20‐hydroxyecdysone (20E) pathways, which, when applied to mosquitoes, successfully disrupts parasite development. Unfortunately, it also impacted mosquito survival and reproductive success (Childs et al., 2016), and will as such be under strong selection pressure for resistance in mosquitoes.

Another idea is to exploit the interaction between mosquito sugar feeding and parasite development. By abundantly providing sugar sources that reduce the vectorial capacity of the mosquito, one may reduce malaria transmission without selective pressure for resistance (Hien et al., 2016). However, abundant nectar availability is predicted to increase behavioural plasticity in daytime or night‐time feeding, possibly due to increased energy availability leading to increased foraging times (Stone et al., 2016). Hence, reduced nectar availability could restrict foraging times and hence push selection more strongly to early evening feeding mosquitoes (behavioural resistance) than in areas with abundant nectar sources.

An additional transmission blocking method is based on the maternally transmitted symbiotic bacteria of *Wolbachia* spp. which results in the mosquito resisting *P. falciparum* infections (Bian et al., 2013; Hughes, Koga, Xue, Fukatsu, & Rasgon, 2011), though it potentially also increases mortality in the mosquitoes.

Although the advantage of such transmission blocking tools is less selection for resistance in the vector, all of the above‐described approaches are of course under selection from the parasite point of view.

A more recent evolutionary‐inspired idea to protect insecticides from resistance evolution is to combine a—still functional—insecticide with a spatial repellent. Repellents have been predicted to delay insecticide resistance evolution (Birget & Koella, 2015). Selective pressure will be for those mosquitoes being deterred by the spatial repellent and are therefore avoiding (lethal) exposure to the insecticide. As a consequence, this method would protect the insecticide from resistance evolution by decreasing the amount of mosquitoes being exposed, as well as driving selection towards increased efficacy of the spatial repellent, ensuring that mosquitoes that are being effectively repelled have a survival advantage (Lynch & Boots, 2016). It has indeed been shown with a population genetics model that the repellent properties of insecticide‐treated bed nets have contributed to a slower evolution of insecticide resistance (Birget & Koella, 2015). Of note is the friction between community and individual protection when using repellents: repellency has a clear benefit for the individual user, but does not provide community protection and thus needs high coverage to be an effective intervention.

For most of the above‐described hypotheses, as for the more conventional ideas of mosaic, mixture and rotation applications, the underlying principles to retard resistance evolution are clear, but solid experimental (especially field‐generated) evidence is lacking.

### 3.2 Drug resistance management strategies

The strategy to deal with the emerging ACT resistance in the Greater Mekong Subregion is to rapidly eliminate all malaria parasites in the affected area (Dondorp, Smithuis, Woodrow, & von Seidlein, 2017; Maude et al., 2009), and the WHO aims to interrupt transmission of multidrug‐resistant *P. falciparum* by no later than 2020 (World Health Organization, 2015c). The question is of course how to eradicate malaria in the face of resistance evolution with the current set of tools as novel drug formulations are not yet available. The success of the proposed strategy depends to a large extent on the very drug the parasites are evolving resistance to. Proposed strategies are (i) drug rotations with existing ACTs such as DHA–PPQ (currently failing) and artesunate–mefloquine (currently effective), (ii) triple drug combinations such as DHA–PPQ–mefloquine, (iii) extension of the treatment course from 3 to 7 days or (iv) using the compound arterolane–piperaquine which is new to the GMS (Dondorp et al., 2017). Major restriction to all these proposed strategies is that they all depend on one or more compounds to which resistance is already prevalent and their continued use allows for the further spread of resistant mutants, which may hamper worldwide malaria elimination efforts. Again the question is as follows: Can evolutionary principles be applied to allow for the most effective resistance management strategies?

Similar to insecticide resistance management strategies, large heterogeneity in drug exposure can be created in space (host mosaic) or time (drug rotation) or by deploying different compounds simultaneously (mixed treatment). ACT rotation and triple mixed treatments are indeed being proposed as potential options for the GMS (see above), although monotherapy is still being deployed for IPTp (SP monotherapy) or severe malaria treatment (artesunate monotherapy during the first 24 hr) (World Health Organization, 2015b). Better resistance management is achieved if drugs can be combined that select for alternative allelic versions of the target locus (Volkman et al., 2017).

Experimental and field data on the optimal resistance management strategy for malaria treatment are absent. However, several population genetic models have predicted that mosaic treatment (or “multiple first‐line treatments” as it is called for antimalarial treatment) is a viable option to retard antimalarial resistance evolution (Antao & Hastings, 2012; Boni, Smith, & Laxminarayan, 2008; Boni, White, & Baird, 2016; Smith, Klein, McKenzie, & Laxminarayan, 2010). In addition, a micro‐simulation on individual level showed that mosaic‐type treatment performed better in delaying resistance emergence and slowed the spread of mutants compared to 5 years of drug rotations or switching drugs following treatment failure (Nguyen et al., 2015). The relative performance in suppressing resistance evolution between mixed treatment strategies (e.g., triple mixed treatments) and mosaic treatment has, to our knowledge, not yet been assessed. Despite these theoretical studies supporting the use of multiple first‐line treatments compared to a single ACT, this has not led to any policy changes to date, reflecting the challenge of translating theory into practice and policy.

Another feature of current drug treatment regimens is that treatment dosages are designed to be sufficiently high to kill the parasites as quickly as possible. This dogma of aggressive chemotherapy was challenged in a series of experiments using rodent malaria parasites which showed that more prudent drug dosages better prevent the evolution of resistance, while still providing clinical benefit (Huijben et al., 2010, 2013; Read, Day, & Huijben, 2011; Wargo, Huijben, de Roode, Shepherd, & Read, 2007). The rationale here is that resistant mutants are competing with intrinsically fitter susceptible wild‐type parasites. This competition slows, or even reverts, the spread of resistance in the absence of treatment. Aggressive treatment rapidly removes this suppressive competition and allows for the proliferation of resistant mutants, arguing for the use of the lowest clinically useful dose to provide the least amount of selective pressure for resistance as necessary (Day & Read, 2016). Similar strategies have been proposed to contain resistance evolution for the treatment of cancer (Gatenby, 2009) and antibiotics (Llewelyn et al., 2017).

### 3.3 Alternative interventions to manage resistance

So far we discussed the use of our front‐line interventions to target the mosquito vector (LLINs and IRS) or the malaria parasite (drugs). Although the majority of novel malaria technologies to control the vector (such as the next generation of LLINs and IRS, spatial repellents, toxic durable wall liners, insecticidal paint, attractive toxic sugar bait, outdoor barrier sprays, toxic barrier sprays and impregnated clothing) and the parasite (new and even triple drug combinations) are still based on insecticides and drugs, respectively, the best method of retarding resistance evolution is to limit—or better avoid—the use of such compounds.

For vector control, several alternative interventions that target (i) the mosquito immatures with natural enemies, bio‐larvicides or other environmental interventions, such as temporary or permanent removal of larval habitats (World Health Organization, 2013), and (ii) the adult vector by house improvement (Tusting et al., 2017), mosquito removal from the population by odour‐baited trapping systems (Homan et al., 2016), increasing the proximity to livestock or using endectocides (when zoophilic vectors are present) (Chaccour & Killeen, 2016) are already implementable (World Health Organization, 2012b) and could be used in combination with insecticides to slow down the spread of resistance. Other techniques, such as genetically (e.g., CRISPR‐Cas9 or irradiation) or biologically modified (e.g., Wolbachia‐infected) mosquitoes, are also being developed, but most are still in earlier stages of development.

In the case of parasite control, fewer alternatives exist. Clearly, an effective malaria vaccine would be a powerful tool in resistance management, as it would not discriminate at any antimalarial target site and additionally reduces the need for antimalarial treatment. Although there are several vaccine candidates at various stages of development, an effective vaccine is only expected post‐2025 (Hemingway, Shretta et al., 2016; Mordmüller et al., 2017; RTS,S Clinical Trials Partnership, 2014; Sissoko et al., 2017). If drugs do need to be used, selection of resistant mutants is still an evolutionary dead end if these mutants cannot spread. As such, rolling out MDA campaigns during the dry season and/or in combination with highly effective vector control reduces the likelihood of onward transmission and hence resistance evolution.

## 4 WILL DIFFERENT TOOLS FACE THE SAME PROBLEMS?

The emergence and spread of resistant mosquitoes is inevitable if we use novel insecticide‐based vector control tools in the same way as we have used insecticide‐based tools to date. If pressure is sufficiently high, and without proper management strategies in place, history will repeat itself. This is true for those intervention whereby there is a direct insecticide–vector contact, but also for those interventions whereby mosquitoes are repelled by a chemical (Stanczyk, Brookfield, Ignell, Logan, & Field, 2010, 2010). Other tools such as endectocides (not yet reported in Anopheles, but shown for *Onchocerca volvulus* (Osei‐Atweneboana et al., 2011)) and genetically modified mosquitoes (Unckless, Clark, & Messer, 2017) are also prone to the development of resistance. In addition, we need to realize that resistance may not necessarily occur through genetic changes, but may be a result of changes in mosquito behaviour.

If we are to target the parasites with a vaccine, we have to realize that even vaccines are not immune to resistance evolution (Barclay et al., 2012; Kennedy & Read, 2017). In addition, evidence suggests that vaccines that do not prevent transmission can create conditions that promote the emergence and spread of highly virulent pathogens strains that cause more severe disease in unvaccinated hosts (Barclay et al., 2012; Read et al., 2015).

## 5 SHOULD WE EXPECT SOME UNFORESEEN INTELLIGENCE IN MOSQUITOES AND PARASITES?

Before we conclude this review, we would like to hypothesize about other, yet unidentified, evolutionary consequences of the way we currently target mosquito and parasite populations, particularly in the face of malaria elimination strategies.

The fact that recent years have seen malaria detection failures (Gamboa et al., 2010; Kozycki et al., 2017) indicates that we may be selecting for parasites that are able to avoid treatment, and as a result increase their survival probability and hence onward transmission. Selection will be extremely intense in those areas aiming at malaria elimination by means of mass screening and treatment: with a sufficiently high coverage, the majority of ongoing transmission after a few rounds of MSAT could be caused by those parasites that have either survived the drug dose or have avoided detection. Such avoidance of detection can be achieved through gene deletion as already observed, but another hypothetical “shelter” may be to find refuge in those special populations excluded from mass treatment: certain age groups (such as infants and elderly) or women in first trimester of pregnancy. So, will we select for parasites that can hide in those special populations that are already difficult to treat?

With the envisioned improvements of the current surveillance systems and the absence of mass drug adminstration, will we select for parasites that hide and survive in asymptomatic individuals (i.e., infected individuals who show no symptoms or are below detection threshold), as we will only detect and treat the symptomatic individuals? Hence, could we be selecting for less virulent parasites to avoid detection (but see Birget, Greischar, Reece, & Mideo, 2017)? There is a clear need for a better understanding of the role of submicroscopic infections in the epidemiology of resistant mutants (Abdul‐Ghani, Mahdy, Beier, & Basco, 2017) as well as the mosquito vector (Mharakurwa et al., 2011). In addition, when we bring more drugs onto the market with a long prophylactic effect, could we select for falciparum parasites with hypnozoite‐like stages that can remain dormant in the liver (as with vivax and ovale malaria)?

And will parasites be able to manipulate their mosquito host to increase transmission success? LLINs and IRS (as well as several novel insecticide‐based vector control tools) are indoor interventions, which target indoor biting and/or resting mosquitoes that prefer to feed on humans. The current selective pressure on mosquitoes can drive them towards outdoor biting (see e.g., Reddy et al. (2011)) and possibly change their host preference to animal biting, especially if they already express an opportunistic feeding behaviour (e.g., *An. arabiensis*). Yet the selective advantage for malaria parasites is maximized by being transmitted from human to human. If mosquito host‐feeding preferences change due to our interventions, will parasite evolution change in response? Will malaria parasites manipulate their mosquito host to maximize their fitness, similar to, for example, *Toxoplasma gondii* parasites manipulating their rodent intermediate host or the fungi within the genus Ophiocordyceps manipulating their ant host (Adamo & Webster, 2013; de Bekker et al., 2014)? There is some evidence pointing in this direction as infected mosquitoes appear to be preferentially attracted to human hosts (Smallegange et al., 2013). With this phenotype already observed in the population, the potential for selection on increased host manipulation is present (discussed by Lefevre et al. 2017).

Further potential secondary evolutionary adaptations may be parasites adapting to insecticide‐resistant mosquitoes with a shorter lifespan by evolving faster development rates, or insecticide‐resistant mosquitoes having a selective advantage in areas of high parasite pressure if the survival cost associated with parasite burden is smaller in insecticide‐resistant mosquitoes (Alout et al., 2016; Rivero et al., 2010). Equally unknown is the role of the mosquito vector in the spread of antimalarial resistance with markedly different resistance profiles observed in human and vector (Mharakurwa et al., 2011). Although it is important to consider all these (and other) possibilities at all times, evolutionary consequences in this complex system (we have to consider the human host, and several species of both mosquito and parasite) are very difficult to predict as a change in one part of the system that reduces fitness in another is likely to lead to an evolutionary response as all components are linked.

## 6 CONCLUDING REMARKS

Antimalarial drugs and insecticides have been used on a wide scale for many decades and will remain cornerstones in malaria elimination efforts. However, resistance has emerged against nearly every antimalarial drug and insecticide on the market. Clinical failure of the current first‐line treatment against falciparum malaria, ACTs, has recently been observed in the Greater Mekong Subregion. There is no alternative treatment available to replace them, which is alarming, especially if ACT resistance will jump to or emerge in sub‐Saharan Africa. The situation for the vector control landscape is not more optimistic, especially as monotherapy is still the norm. Hemingway and colleagues already warned us last year: “With no new insecticide class to replace the pyrethroids expected for a decade, the threat of resistance derailing malaria control has become an issue of urgency that can no longer be ignored without risking a global public health catastrophe” (Hemingway, Ranson et al., 2016). A greater understanding of the general evolutionary principles that are at the core of emerging resistance is urgently needed, and will allow us to develop improved resistance management strategies for both drugs and insecticides, as has been argued in various other disciplines, including herbicide (Neve, 2007) and cancer resistance management (Enriquez‐Navas, Wojtkowiak, & Gatenby, 2015). This involves a greater investment in modelling evolutionary dynamics and performing laboratory and (semi)field trials to develop and test novel resistance management strategies (which include techniques such as mosaics, rotations, mixtures, refugees and/or the combination of various (non)chemical interventions). Yet, despite being an evolutionary problem at its core, evolutionary biologists themselves have been largely ignoring the resistance problem (Read & Huijben, 2009). An additional obstacle is that when it comes to evolutionary principles, there is a general disconnect between the academic world and policy makers, even though malaria programme managers and academics both acknowledge that the evolution of pathogen and vector is hampering our progress of malaria control and eradication and that interventions may fail if evolutionary biology of the disease is disregarded (Ocampo & Booth, 2016). Therefore, we should think ahead, assume evolution will happen and wear our evolutionary hat at all times when rolling out existing and new interventions.
